# Assembling Surface Linker Chemistry with Minimization of Non-Specific Adsorption on Biosensor Materials

**DOI:** 10.3390/ma14020472

**Published:** 2021-01-19

**Authors:** Jack Chih-Chieh Sheng, Brian De La Franier, Michael Thompson

**Affiliations:** Department of Chemistry, University of Toronto, 80 St. George Street, Toronto, ON M5S 3H6, Canada; jack.sheng@mail.utoronto.ca (J.C.-C.S.); brian.delafranier@utoronto.ca (B.D.L.F.)

**Keywords:** biosensor surface linker, antifouling chemistry, acoustic wave detection

## Abstract

The operation of biosensors requires surfaces that are both highly specific towards the target analyte and that are minimally subject to fouling by species present in a biological fluid. In this work, we further examined the thiosulfonate-based linker in order to construct robust and durable self-assembling monolayers (SAMs) onto hydroxylated surfaces such as silica. These SAMs are capable of the chemoselective immobilization of thiol-containing probes (for analytes) under aqueous conditions in a single, straightforward, reliable, and coupling-free manner. The efficacy of the method was assessed through implementation as a biosensing interface for an ultra-high frequency acoustic wave device dedicated to the detection of avidin via attached biotin. Fouling was assessed via introduction of interfering bovine serum albumin (BSA), IgG antibody, or goat serum. Improvements were investigated systematically through the incorporation of an oligoethylene glycol backbone employed together with a self-assembling diluent without a functional distal group. This work demonstrates that the incorporation of a diluent of relatively short length is crucial for the reduction of fouling. Included in this work is a comparison of the surface attachment of the linker to Si_3_N_4_ and AlN, both materials used in sensor technology.

## 1. Introduction

Interest has been shown over the years in the application of biosensor technology in a variety of fields such as environmental and food analysis, biopharmaceutical research, biodefence, and clinical diagnostics [1]. This is due to the fact that such devices potentially possess the capability for the selective, real-time and label-free detection of analytical targets in a specific sample. The fabrication of a particular device depends heavily on the immobilization of the biosensing element (a probe such as an antibody, aptamer, etc.) onto the transducer material in a controlled manner in order to produce a reproducible and stable configuration where the biological activity of the biosensing element is preserved [2]. Attachment of probes to the various materials used in biosensor technology has involved a number of different strategies. These include covalent binding often via linking chemistry such as the ubiquitous 1–ethyl–3–[3-dimethylaminopropyl] carbodiimide hydrochloride–N–hydroxysulfosuccinimide (EDC–NHS) system [3,4], gel entrapment or encapsulation [5], molecular imprinted polymers [6], Langmuir–Blodgett deposition technology [7,8], and self-assembled monolayers (SAMs) [9,10].

In addition to probe attachment, the biosensing interface must feature high selectivity and sensitivity towards the target analyte, even in the presence of unwanted species. Such fouling or non-specific adsorption of device surfaces by interfering proteins and other moieties in blood, serum, urine, and cerebrospinal and interstitial fluid has for a number of years constituted an Achilles heel with respect to applications of biosensor technology, especially in the area of clinical biochemistry [11]. This issue has spawned the development of several approaches for reducing the fouling of devices, which, unsurprisingly, have a great deal in common with surface chemistries employed in the world of biocompatibility research [12,13,14]. These include amino acids, peptides and peptoids, poly(ethylene glycol)-based coatings, zwitterionic monolayers, and carbohydrate derivatives [15].

Surface modification of materials used for biosensor construction should ideally combine both the possibility of a linking system for the probe with antifouling chemistry in a truly tandem fashion. SAM modifications provide a simple and robust way to achieve this. Many SAM chemistries are very stable, and it is typically easy and low cost to apply them to surfaces. In the case of SAMs, the biosensing element is also typically bound very close to the sensor surface, which improves the signal achieved in flat sensors such as those based on acoustic waves.

**Figure 1:**
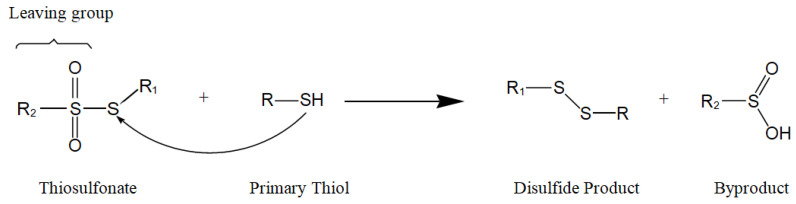
General mechanism of the reaction between a thiosulfonate and a thiol.

When designing SAM linkers, there are three essential and distinct parts to consider, namely the tail function, the head group, and the backbone. The tail function is used to anchor the linker itself onto the surface of the transducer, while the head group is able to immobilize the biosensing element onto the SAM. The long backbone provides the linker with the necessary intermolecular forces to form a stable assembly. In this work, the well-established trichlorosilyl group was utilized to attach an assembling linker to the hydroxylated substrate of choice [16]. The thiosulfonate head group was employed to attach biotinthiol covalently via disulfide bond formation [17]. In this chemistry (Figure 1), the reacting thiosulfonate is composed of a residue R_1_ attached to an electrophilic sulfur atom carrying a sulfinyl leaving group with residue R_2_. A thiol moiety, in this case biotinthiol, forms a disulfide bond with the electrophilic sulfur atom.

The backbone of a linker plays a crucial role in the formation of SAMs. In general, linkers consisting of long alkyl backbones (8 to 18 carbons in length) are able to form crystalline-like rigid monolayer assemblies, where the alkyl backbones are densely packed [18]. Those with longer alkyl chains consistently form more ordered layers than those formed from shorter chains [19]. Films with ethylene glycol chains in both forms, i.e., poly(ethylene glycol) (PEG) and oligo(ethylene glycol) (OEG), have for some time been known to resist non-specific adsorption [20,21,22,23]. The mechanism behind the “PEG” effect has been the subject of debate over time to the present day. Entropy repulsion and the presence of a so-called “water barrier” have often been separately proposed to explain the effect [24,25,26,27,28]. With regard to entropic repulsion, adsorption of biomolecules on the surface is thought to cause the glycol chains to compress. The resulting volume restriction and/or osmotic repulsion leads to a loss in configurational entropy. Thus, the entropic repulsion force repels the approach of biomolecules that have low affinity for the surface, as their fouling of the PEG layer would further compress it, thereby reducing surface entropy. In contrast, the water barrier mechanism is attributed to oxygen atoms within the ethylene glycol chain hydrogen bonding with water molecules in solution. This structure supposedly forms a physical barrier made of hydrogen bonded water molecules, which prevents direct contact between non-specific biomolecules and the surface by masking the surface as part of the bulk aqueous solution.

In our research, we have shown that an ultra-thin (0.6 nm on silica) SAM with a distal –OH group containing a single glycol entity is highly effective in the reduction of fouling from undiluted goat serum [29]. Apparently, the hydrated –OH group functions in concert with the hydrated glycol group in producing this effect. The modified surface is also highly efficacious in the significant reduction of platelet aggregation from blood [30,31,32]. None of this research included an investigation of the influence of chain length or number of glycol moieties, which is one of the goals of the present paper. A second aim was to employ the molecule (termed a diluent) mentioned above to space out linker molecules in a deliberately mixed SAM. The purpose of this system is to relieve steric hindrance around the head function in order to facilitate probe attachment together with the combined possibility of reducing non-specific adsorption. Such an arrangement can also assist in retaining the integrity of attached probes (e.g., a protein molecule) [33]. A preliminary study such as this has been performed, but this did not involve testing the surface chemistry with protein-containing samples, or with goat serum as a source of interference [34]. We report such an investigation in the present paper.

The antifouling ability of various lengths of oligoethylene glycol layers, as well as their diluted versions, was evaluated using a high frequency acoustic wave device known as an EMPAS [35]. This device uses a copper coil to drive a thin quartz crystal at a high harmonic frequency in order to generate a signal greater than 800 MHz. This allows for very sensitive measurements due to a decrease in the signal-to-noise ratio at these high frequencies. The ability to provide antifouling in these sensors greatly increases the signal-to-noise ratio, allowing for better and more sensitive detection of the desired species. Investigating the effect of chain length, and dilution of glycol-based antifouling layers will enable us to better understand the mechanisms of their antifouling ability, as well as to design better layers to improve biosensors.

These surface modifications were also extended to aluminum nitride (AlN surfaces), which is another material used in acoustic wave devices [36], and to Si_3_N_4_, which is a material used in cantilever devices where antifouling is also crucial [37]. The antifouling ability of layers on these devices was not evaluated in the present work.

## 2. Materials and Methods

### 2.1. Materials and Surface-Modifying Molecules

Anhydrous solvents (THF, MeCN, CH_2_Cl_2_, CHCl_3_, PhMe, Et_2_O) were used throughout this work. Freshly distilled anhydrous MeOH, EtOH, and Et_3_N (from KOH) were systematically used for biotinthiol immobilization. Avidin (from egg white, lyophilized powder), IgG from rabbit, bovine serum albumin (BSA), goat serum, and Dulbecco’s phosphate buffered saline (PBS) were purchased from Sigma-Aldrich^®^ (St. Louis, MO, USA). Unless otherwise noted, other chemicals were also purchased from Sigma-Aldrich^®^ and used as received. Quartz crystals (AT-cut, 13.5 mm in diameter, 20 MHz fundamental frequency) were purchased from Lap-Tech Inc^®^ (York, SC, USA). EMPAS measurements were performed at 864 MHz (43rd harmonic). SAM formation and biotinthiol immobilization were performed in a glovebox maintained under an inert (N_2_) and anhydrous atmosphere. The crystals were systematically handled with thoroughly pre-cleaned stainless steel tweezers in order to minimize any external contamination. ^1^H and ^13^C NMR spectra were recorded at room temperature on a Varian Mercury 300 MHz (Camcor, Burlington, NC, USA) or a Mercury 400 MHz spectrometer using CDCl_3_ and CD_3_OD as the NMR solvent. ^1^H and ^13^C NMR spectra are referenced to the residual solvent peak (CDCl_3_: 7.27 and 77.23 ppm; CD_3_OD: 3.31 ppm). Chemical shifts are given in ppm.

**Figure 2:**
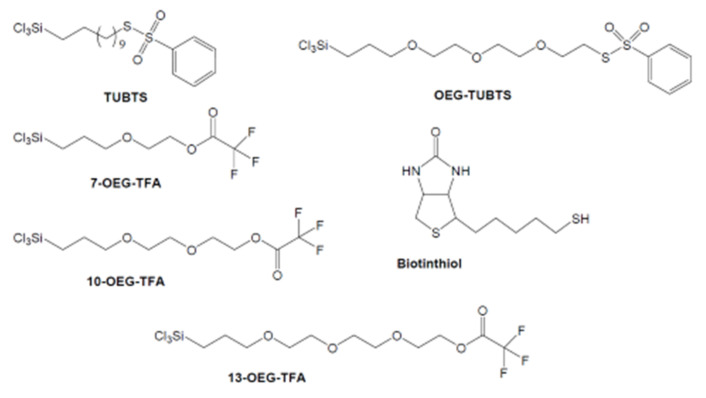
Structures of S–(11–trichlorosilyl-undecenyl) benzenethiosulfonate (TUBTS), S–(2–(2–(2–(3–trichlorosilyl–propyloxy)–ethoxy)–ethoxy)–ethyl) benzenethiosulfonate (OEG–TUBTS), trifluoroacetic acid 2–(3–trichlorosilyl–propyloxy)–ethyl ester (7–OEG–TFA), trifluoroacetic acid 2–(2–(3–trichlorosilyl–propyloxy)–ethoxy)–ethyl ester (10–OEG–TFA), trifluoroacetic acid 2–(2–(2–(3–trichlorosilyl-propyloxy)–ethoxy)––ethoxy)–ethyl ester (13–OEG–TFA), and biotinthiol.

The synthesis of S–(11–trichlorosilyl-undecenyl) benzenethiosulfonate (TUBTS), S–(2–(2–(2–(3–trichlorosilyl–propyloxy)–ethoxy)–ethoxy)–ethyl) benzenethiosulfonate (OEG–TUBTS), trifluoroacetic acid 2–(2–(3–trichlorosilyl–propyloxy)–ethoxy)–ethyl ester (10–OEG–TFA), trifluoroacetic acid 2–(3–trichlorosilyl–propyloxy)–ethyl ester (7–OEG–TFA), and trifluoroacetic acid 2–(2–(2–(3–trichlorosilyl-propyloxy)–ethoxy)––ethoxy)–ethyl ester (13–OEG–TFA), and biotinthiol were available. All molecules are shown in Figure 2, with synthetic procedures and attendant characterization data being detailed in Supplementary Materials.

### 2.2. Substrate Preparation

Quartz crystals were first sonicated in 20 mL of soap for 30 min. The crystals were successively rinsed with hot water followed by distilled water, and then gently dried with forced air followed by soaking in 6 mL of piranha solution pre-heated to 90 °C for 45 min using a hot water bath. The crystals were then rinsed three times with distilled water and three times with spectro-grade methanol. Next, the substrates were sonicated in methanol for 2 min before being individually stored into vials and placed in an oven maintained at 150 °C for drying. After 2 h, the crystals were transferred into a 60%-maintained (MgNO_3_·6H_2_O) humidity chamber for 24 h. AlN and Si_3_N_4_ substrates were first sonicated in 20 mL of soap for 30 min. The substrates were successively rinsed with hot water followed by distilled water. Then, they were rinsed three times with deionized water. Finally, they were rinsed three times in acetone and sonicated for 2 min, also in acetone. This process was repeated with ethanol and then methanol. The substrates were then dried with a gentle stream of nitrogen gas and plasma cleared for 5 min.

### 2.3. Silanization Procedure

All glassware was pre-treated with an octadecyltrichlorosilane (1/20 (*v*/*v*)) solution in anhydrous toluene overnight to prevent any undesired reactions of our trichlorosilyl linker/diluent with the walls of the glassware during the silanization procedure. For pure SAMs, neat linker (TUBTS or OEG–TUBTS, 10 μL) or neat diluent (7–OEG, 10–OEG, or 13–OEG, 10 μL) was first diluted with anhydrous toluene (10 mL). The resulting solution was portioned (1 mL) into test tubes in which cleaned substrate was soaked. The test tubes were then sealed and set aside for 1 h. Afterwards, the substrate was rinsed twice with dry toluene, and then sonicated for 2 min in another portion of toluene. After a final rinsing with toluene, the above procedure was repeated with dry chloroform. Finally, the crystals were gently dried under forced air, and then individually transferred into vials for storage or immediately engaged in the subsequent biosensing element immobilization.

For 1:1 mixed SAMs, a combination of two neat linkers/diluents (OEG–TUBT, 7–OEG, 10–OEG, or 13–OEG), 5 μL each, was diluted with anhydrous toluene (10 mL). The resulting solution was portioned (1 mL) into test tubes in which cleaned substrate (quartz crystal, TSM crystal, or AlN or Si_3_N_4_ substrates) were soaked. The test tubes were then sealed and set aside for 1 h. Afterwards, the substrates were rinsed twice with dry toluene, and then sonicated for 2 min in another portion of toluene. After a final rinsing with toluene, the above rinsing steps were repeated with dry chloroform. Finally, the substrates were gently dried under forced air, and then individually transferred into vials for storage or immediately engaged in the subsequent biosensing element immobilization.

### 2.4. Attachment of Biotinthiol

Biotinthiol (6.0 mg) was dissolved into a solution of 1:1 deionized water and methanol (6.0 mL). Et_3_N (6.0 μL) was also added to this solution. The resulting solution (1.0 mg/mL) was portioned (1 mL) into dry test tubes, in which the freshly prepared SAM-coated substrate was soaked. The test tubes were then sealed and set aside for 1 h. Afterwards, the substrate was rinsed three times with spectro-grade methanol, then sonicated for 2 min in spectro-grade methanol, followed by another rinse of spectro-grade methanol. Finally, the substrate was gently dried under a stream of nitrogen gas and transferred into individual vials for storage.

**Figure 3:**
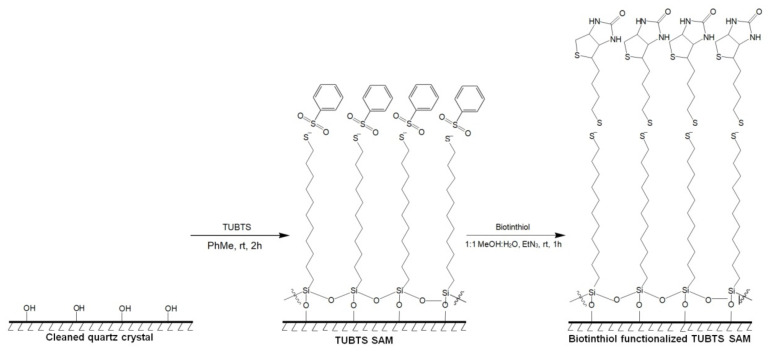
Surface attachment of TUBTS to quartz followed by immobilization of biotinthiol.

The attachment of the basic TUBTS molecule with subsequent immobilization of biotinthiol is depicted in Figure 3. The protocol for the glycol-containing version is analogous to this method.

### 2.5. Cleavage of the Diluent TFA Head Group

**Figure 4:**
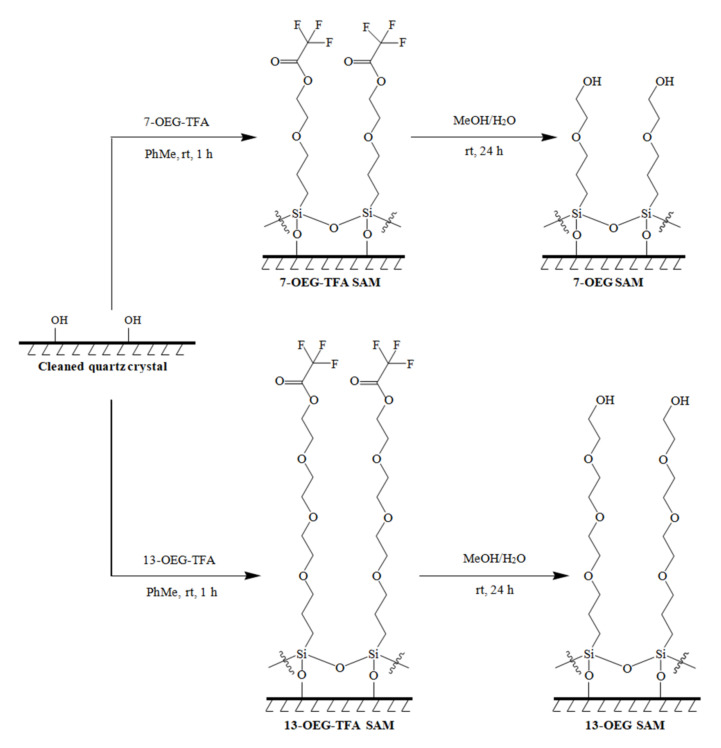
Surface attachment of 7–OEG–TFA and 13–OEG–TFA followed by cleavage of the protecting TFA Group. An identical protocol was employed for 10–OEG–TFA.

A solution of 1:1 deionized water and methanol was portioned (1 mL) into dry test tubes in which the freshly prepared SAM-coated substrate was soaked. The test tubes were then sealed and set aside for 1 h. Afterwards, the substrate was rinsed three times with spectro-grade methanol, and then sonicated in spectro-grade methanol for 2 min followed by another rinse of spectro-grade methanol. Finally, the substrate was gently dried under a stream of nitrogen gas. The substrate was transferred into individual vials for storage. The surface chemistry for this step is shown in Figure 4.

### 2.6. Surface Characterization

Substrate contact angles (CAs) were measured with the KSV contact angle measurement (CAM) system (KSV Instruments Ltd., CAM101, Espo, Finland) and ultrapure water was used as the test liquid. Once the droplets were gently deposited onto the surfaces, they were allowed to settle for 10 s. Five frames were then recorded at 1 s intervals.

X-ray photoelectron spectroscopic (XPS) analysis was performed with the Thermo Scientific K-Alpha XPS spectrometer (ThermoFisher, East Grinstead, UK), located at Surface Interface Ontario at the University of Toronto in Toronto, ON, Canada. The samples were analyzed with an Al Kα X-ray source, at take-off angles of 20° relative to the surface. The binding energy scale was calibrated to the main C(1 s) signal at 285.0 eV. Peak fitting and data analysis were performed using software provided with the instrument.

### 2.7. EMPAS Measurements

AT-cut 20.0 MHz quartz crystals were placed in a flow-through system. One side of the crystal was exposed to liquid, and the other side was exposed to air. Runs were performed with the crystal in the horizontal position and at ambient temperature. The crystal was secured in the holder using an O-ring. A computer equipped with LabView 6.0 controlled the radio frequency generator and lock-in amplifier, while acquiring data during the course of the measurement.

Avidin or BSA buffer stock solutions (1 mg/mL) were prepared by dissolving 2.0 mg of solid avidin or BSA into 2.0 mL of PBS buffer. Avidin buffer sample solutions (0.001 mg/mL to 0.2 mg/mL) were prepared by serial dilutions from the stock solution with PBS buffer. BSA buffer sample solution (0.1 mg/mL) was prepared by adding 90 μL of PBS buffer and 10 μL of BSA buffer stock solution. IgG sample solution (0.1 mg/mL) was prepared by diluting an IgG solution as purchased with the correct volume of PBS. Goat serum sample solution was used without modification or dilution.

Each slide was thoroughly flushed with PBS at a rate of 50 μL/min using a syringe pump. After ensuring uniform coverage of the disc with PBS (i.e., no bubbles), the resonant frequency of the disc was manually found using an oscilloscope. This was achieved by plugging the cable from the lock-in amplifier into the oscilloscope and then manually scanning frequencies to find the 43rd harmonic (0.863 GHz). This frequency was then inputted into the LabView program to be used as the standard during the course of the EMPAS measurement. After initializing the run, the frequency signal was allowed to stabilize for 10–15 min. Then, a 50 µL portion of sample was injected into the flow-through system using a low-pressure chromatography valve. This was followed with the uninterrupted flow of PBS at a rate of 50 μL/min to remove any non-specifically or loosely bound species. PBS was flowed until a stable baseline was achieved (10–15 min). Changes to the resonant frequency were noted during the course of the run. Prior to the introduction of a new slide, the system was vacuumed dry using a peristaltic pump and carefully dried.

## 3. Results and Discussion

### 3.1. Surface Characterization

#### 3.1.1. Contact Angle Measurement

The results for CAM of the various procedures are shown in Table 1.

Both TUBTS and OEG–TUBTS yielded values that increased significantly after silanization on quartz due to the addition of the hydrophobic head group, while in both cases there was a slight decrease following biotinthiol immobilization. This result is associated with the expected introduction of the more hydrophilic moiety, biotinthiol. This suggested that the OEG–TUBTS SAM was successfully formed on the quartz crystal and biotinthiol was subsequently immobilized onto the SAM. In addition, the CAM values of OEG-TUBTS SAM (72° ± 4°) and biotinthiol-functionalized OEG–TUBTS SAM (64° ± 3°) were lower than their counterparts in TUBTS SAM formation (96° ± 3° and 78° ± 3°), respectively. This could be explained by the presence of oxygen atoms in the backbone of OEG–TUBTS, which renders the SAM less hydrophobic.

The CAM values for the proposed surface diluents (10–OEG–TFA, 7–OEG–TFA, and 13–OEG–TFA) are all increased as anticipated due to the inclusion of the glycol chain, but reduced dramatically on cleavage of the TFA group to the hydrophilic –OH moiety. The usual trends for silanization and subsequent biotinthiol immobilization were observed for the surfaces of the mixed SAMs. In addition, the CAM values of OEG–TUBTS/7–OEG-TFA SAM (64° ± 4°) were lower than those of OEG–TUBTS (72° ± 4°). This could be explained by the diluent spacing out the linker, which effectively exposed more OEG backbone and made the surface less hydrophobic. Very similar trends are observed for the surface modification of the Si and Al nitrides.

#### 3.1.2. XPS Analysis

In summary, the XPS analysis (Table 2) shows the overall reduction in Si and O relative atomic percentages as a result of coverage afforded by the various modifying entities. As expected, the presence of sulfur is indicated for all the TUBTS-modified surfaces, with the observation of two peaks for the sulfur signal (not shown here) that are attributed to the two sulfur atoms in different oxidation states (S^0^ and S^IV^) of the TUBTS head function. A new peak appeared in the nitrogen signal after biotinthiol immobilization on the various molecules with the TUBTS head group. This confirms that the expected reaction between the thiol and thiosulfonate to produce a disulfide bond had occurred.

After silanization with the proposed diluents, 10–OEG–TFA, 7–OEG–TFA, and 13-OEG-TFA, the relative atomic percentages of fluorine and carbon increased, whereas the percentages of silicon (%Si) and oxygen slightly decreased. The increases in the former are explained by the deposition of the molecules onto the surface, which added more fluorine and carbon atoms. In addition, the fluorine signals completely disappeared after the diluent SAMs were treated with a solution of methanol and deionized water (3.8% to 0.0%). This confirmed that the trifluoroacetate protecting the TFA head function was cleaved in each of the three cases. It should also be noted that the percentage for silicon after deprotection was highest for 7–OEG, lower for 10–OEG, and lowest for 13–OEG. This suggests that the 13–OEG layer is the thickest, as it more greatly masks the silicon signal, compared to 7–OEG, which is the thinnest layer.

Analogous results to the above were generally observed for the mixed SAMs, given the removal of the TFA functionality being indicated by the disappearance of F and introduction of N being associated with biotin, respectively. Finally, as for quartz, the relative atomic percentages observed on the nitrides confirmed the presence of attachment of TUBTS and eventual addition of biotin. However, the decrease in %O was due to the formation of the SAM over the thin layer of oxidation on the surface of cleaned Si_3_N_4_. Thus, less characteristic atoms of this material and its oxidized layer were seen by XPS surface analysis.

### 3.2. EMPAS Detection of Surface Interactions

#### 3.2.1. TUBTS SAMs

**Figure 5:**
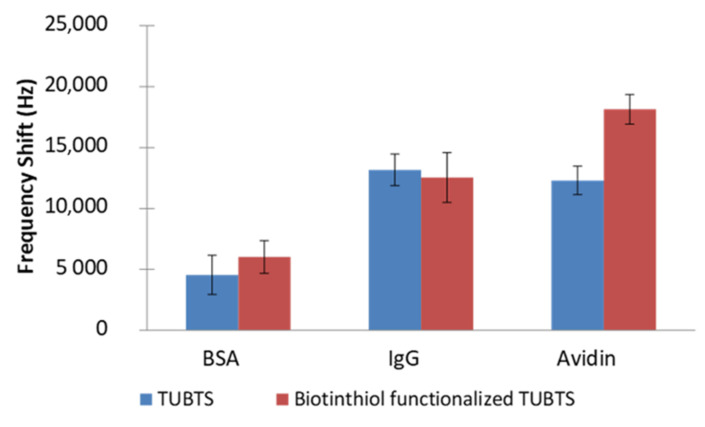
EMPAS frequency shifts measured with TUBTS and biotinylated TUBTS self-assembling monolayers (SAMs), using 0.1 mg mL^−1^ BSA, IgG, and avidin solutions in PBS buffer solution.

The performance of the biosensing interface constructed by TUBTS SAMs was studied with the EMPAS system at an ultra-high resonating frequency of 0.86 GHz (43rd harmonic). Three different proteins (avidin, BSA, and IgG) were used separately in the EMPAS experiments. With each protein, two sets of independent frequency shift measurements were conducted using 0.1 mg mL^−1^ solutions (4 replicates per set) of the appropriate protein in PBS. For the first set, biotinthiol-functionalized TUBTS SAMs were used to examine the interaction of each protein with biotin, which included the expected specific binding of avidin to biotin. For the next set, TUBTS SAMs without biotinthiol modification were used as a control to record non-specific adsorption of all the proteins on the biosensing interface. The results of these experiments for TUBTS are depicted in Figure 5.

There is a notable downward shift in frequency for the interaction of avidin with the biotinylated SAM (18 kHz), but there is also a significant change of 12 kHz for the case that does not involve biotin. Responses to the other two proteins were also observed for both SAMs. Clearly, there is a high-level non-specific adsorption evident in these experiments, which is likely caused by protein interaction with the hydrophobic surface of densely packed TUBTS SAMs (CA 96°).

**Figure 6:**
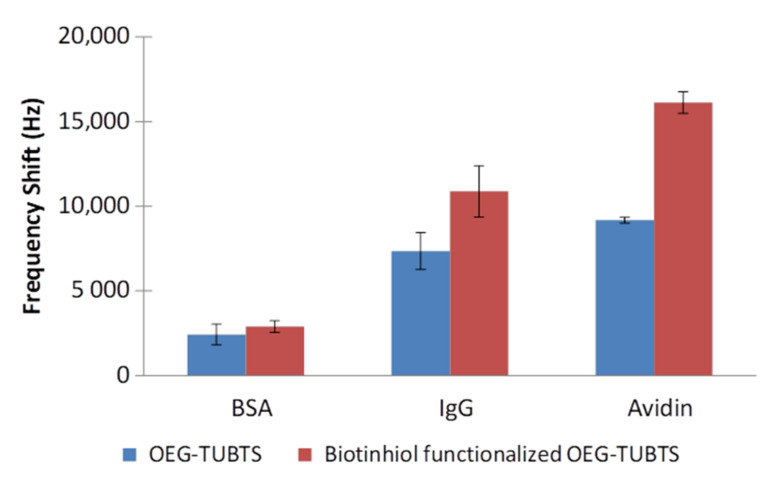
EMPAS measured with OEG–TUBTS and biotinylated OEG–TUBTS SAMs, using 0.1 mg mL^−1^ BSA, IgG, and avidin solutions in PBS buffer solution.

**Figure 7:**
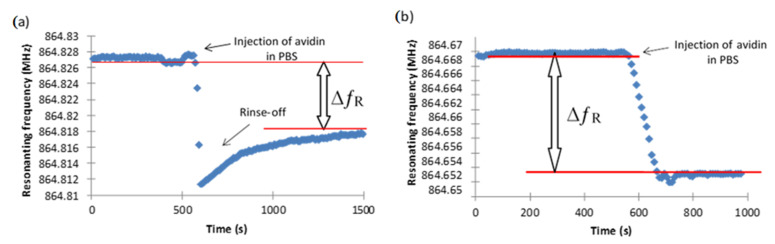
EMPAS response profiles for (**a**) OEG–TUBTS SAM and (**b**) biotinylated OEG–TUBTS SAM using a 0.1 mg mL^−1^ avidin solution.

As noted above, polyethylene glycol was employed ubiquitously to avoid adsorption of biological moieties to surfaces. Accordingly, analogous experiments to those performed with TUBTS were conducted with the version containing a glycol functionality, OEG-TUBTS, and the biotinylated molecule (Figure 6). The addition of the OEG backbone brought only a minor improvement to the performance of the system in that the overall level of non-specific adsorption was reduced by a moderate average of 25% across all the protein responses. It is interesting to note that the EMPAS response profile for non-specific adsorption rises following the frequency drop. This phenomenon is likely due to the fact that loosely bound protein is ultimately rinsed off the surface by the buffer flow. As expected, this effect was not observed for the interaction of avidin with the biotinylated SAM (Figure 7).

The non-specific adsorption observed with these SAMS is again associated with the hydrophobic nature of the monolayer, since the effect of the glycol group will be mitigated by the close-packed nature of the monolayer. This observation led to the decision to include a diluent molecule within the SAM to space out the tightly packed linker in order to further improve the performance of our system.

#### 3.2.2. Potential SAM Diluents

**Figure 8:**
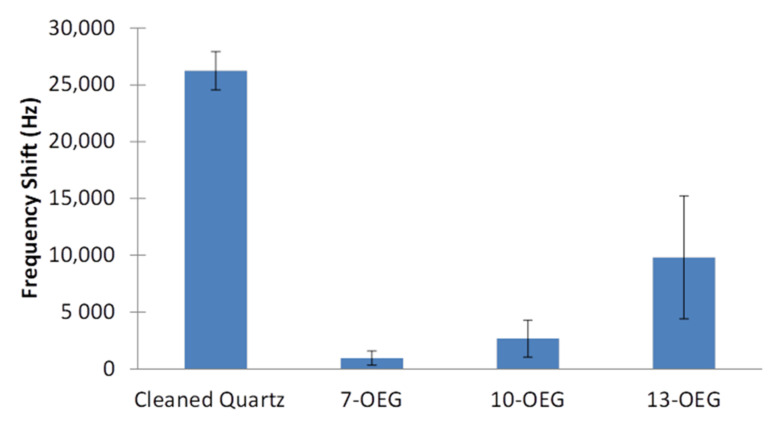
EMPAS non-specific adsorption frequency shifts measured with undiluted goat serum using a cleaned quartz crystal and 7–OEG, 10–OEG, 13–OEG SAMs.

It has already been shown that 7–OEG (TFA group being removed) can substantially reduce adsorption of proteinaceous species to a surface [33] and be employed as a diluent [34]. However, it was considered critical in this work to examine the role of diluent chain length with regard to non-specific adsorption. Accordingly, in addition to 7–OEG–TFA, the sister molecules, namely 10–OEG–TFA and 13–OEG–TFA, were synthesized. These diluents, which vary by the length of their OEG units, were studied by EMPAS experimentation following the usual cleavage of the protecting TFA head group. Figure 8 clearly shows that all three surface-attached molecules to be employed as diluents show a significant reduction (>60%) in EMPAS response to undiluted goat serum, when compared to the result for a cleaned quartz device. Notably, the response obtained with the 7–OEG SAM (950 Hz) was much lower than those found for the longer molecules, i.e., 10–OEG SAM and 13–OEG SAM (2600 and 9800 Hz, respectively). This result strongly suggests that shorter diluents possess enhanced resistance to non-specific adsorption. This is due to the lower packing density formed by shorter molecules where there is an expectation of more disorder. As the chain length increases, the SAM becomes more densely packed, forming a more crystalline-like and rigid assembly, which is predominantly caused by increased hydrophobic forces due to an increased number of CH moieties. As a result, the ethylene glycol chain within the shorter-chain SAM is more solvated by water molecules by penetration than that for the longer versions.

This explanation is supported by both neutron reflectometry [38] and molecular dynamic [39] studies of the 7–OEG system. In both cases, there is a confirmation of extensive penetration of water molecules within the SAM. We believe that this is a key link to the impressive ability of the 7–OEG–SAM in preventing non-specific adsorption. As outlined above, both the water barrier hypothesis, what has been termed the kosmotrophic effect [40], and entropy repulsion mechanisms work in a concerted mechanism to achieve a high degree of resistance to surface non-specific adsorption. It should also be noted that longer chain lengths showed a greater degree of variance in antifouling ability, as can be seen by the higher error in Figure 8 for 13–OEG compared to 10–OEG compared to 7–OEG. This is likely due to greater movement and less stable hydration present at longer chain lengths, increasing the entropy of the layer, which would add greater variance to the antifouling of these layers.

#### 3.2.3. Mixed SAMs

**Figure 9:**
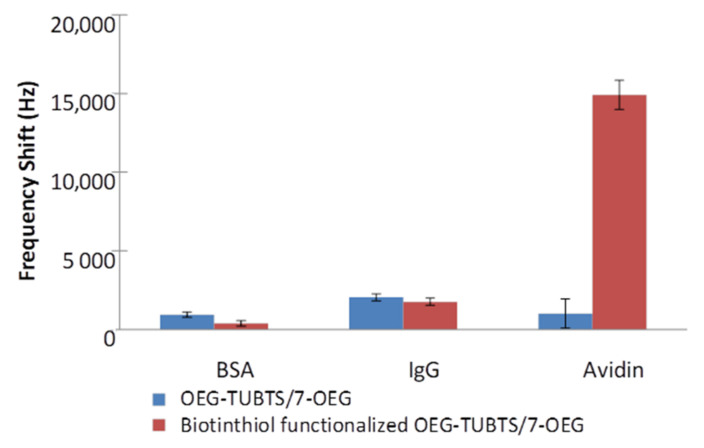
EMPAS frequency shifts measured with OEG–TUBTS/7–OEG and biotinylated OEG–TUBTS/7–OEG SAMs, using 0.1 mg mL^−1^ BSA, IgG, and avidin solutions in PBS buffer solution.

Equivalent EMPAS measurements to those for TUBTS and OEG–TUBTS described above were conducted on a 1:1 molecular ratio for a mixed SAM produced from OEG–TUBTS and 7–OEG. The latter was chosen as the diluent in view of its performance in terms of the prevention of non-specific adsorption outlined in the previous section (with the TFA group removed). The results of these experiments are shown in Figure 9, where it is very evident that incorporation of 7–OEG into the monolayer results in a dramatic reduction of non-specific adsorption. Both responses for BSA and IgG were much lower than for previous cases, as indeed was found for the interaction of non-biotinylated OEG–TUBTS with avidin. This improvement is likely due a combination of the spacing out of the longer linkers, allowing for entropy repulsion, and the inherent ability of 7–OEG in preventing non-specific adsorption. It is also interesting to note that the standard deviation of these responses was exceptionally low (±385 Hz on average). This relative standard deviation (RSD) is very close to the background noise of the EMPAS system (±200 Hz). This was likely due to the strong reduction of random non-specific adsorption.

**Figure 10:**
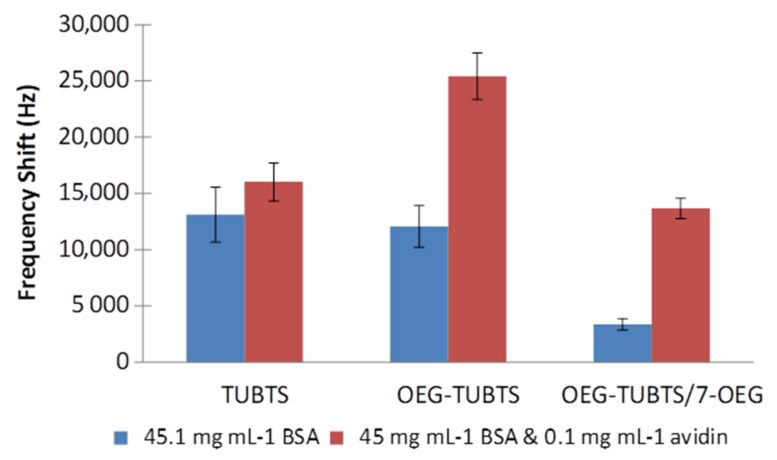
EMPAS frequency shifts measured with 45.1 mg mL^−1^ BSA and 45 mg mL^−1^ BSA with 0.1 mg mL^−1^ avidin solutions in PBS, using biotinylated TUBTS, OEG–TUBTS, and OEG–TUBTS/7–OEG SAMs.

Finally, a study of the effect of a high concentration of BSA was conducted in experiments analogous to those outlined above. In order to mimic serum, a concentration of 45 mg mL^−1^ BSA was employed because serum albumin is the most abundant protein in the blood plasma of mammals (e.g., roughly 34–54 mg mL^−1^ in human blood). Figure 10 shows the EMPAS response for surface-biotinylated TUBTS, OEG–TUBTS, and OEG–TUBTS/7–OEG (mixed) SAMs. As anticipated, there is a significant increase in non-specific adsorption caused by the high concentration of BSA, but encouragingly the mixed monolayer still provides a ratio of 5:1 for avidin signal when exposed to the combined BSA/avidin solution.

## 4. Final Remarks

The results of the present paper confirm that thiosulfonate-based trichlorosilane linkers can be employed to construct robust and durable SAMs onto a hydroxylated surface of various types. The resulting SAMs had the ability to chemoselectively immobilize thiol-containing molecules under aqueous conditions in a single, straightforward, reliable, and coupling-free manner. In new experiments, the biosensing properties of the modified interface in terms of specific and non-specific adsorption were successfully evaluated with EMPAS using avidin, BSA, and IgG in PBS solution. Although the introduction of the glycol group into the linker chain provided a small improvement in terms of reducing non-specific adsorption, the tight molecular packing clearly hindered the effect of the OEG backbone. Experiments with three distinct potential SAM diluents of variable chain length were performed in an attempt to mitigate the packing effect. By far, the most efficacious of these was the short-chain 7–OEG that reduced fouling by the various proteins by 75%, an effect which is ascribed to the allowance of water penetration into the SAM. It is very likely that the longer chain diluents suffered from the same packing issue as for the basic OEG–TUBTS-based SAM, in that such water introduction is prevented. Use of 7–OEG in a mixed SAM of a 1:1 ratio was very successful in reducing non-specific adsorption to a level 8-fold better than for the basic OEG–TUBTS SAM.

The surface modification strategy was also expanded to Si_3_N_4_ and AlN, both materials employed in medical applications. This was successful, although in comparison with the results for silica the indication was that the surface coverage obtained in the study was somewhat less than that for SiO_2_. It is likely that this was caused by a reduced population of –OH groups available for silanization. Unlike the case for silica, more severe oxygenation may be required for the other two materials [36].

## Figures and Tables

**Table 1:** Contact angle (CA) values for modified surfaces.

Surface	Value
Quartz	12° ± 2°
TUBTS	6° ± 3°
TUBTS–Biotin	78° ± 3°
OEG–TUBTS	72° ± 4°
OEG-TUBTS-Biotin	64° ± 3°
10–OEG–TFA	70° ± 2°
10–OEG	25° ± 4°
7–OEG–TFA	66° ± 2°
7–OEG	20° ± 5°
13–OEG–TFA	77° ± 3°
13–OEG	24° ± 3°
OEG–TUBTS/7–OEG–TFA	64° ± 4°
OEG–TUBTS/7–OEG–Biotin	55° ± 6°
	-
Si_3_N_4_	5° ± 2°
Si_3_N_4_/TUBTS	71° ± 3°
Si_3_N_4_/TUBTS–Biotin	63° ± 3°
	-
AlN	4° ± 2°
AlN/TUBTS	81° ± 1°
AlN/TUBTS–Biotin	69° ± 4°

**Table 2:** Relative atomic percentages for elements present on material surface from XPS signals obtained at 20° angle relative to the surface ^1^.

Surface	Si (2p)	O (1s)	C (1s)	S (2p)	N (1s)	F (1s)
Quartz	40.3	52.8	6.9	0.0		
TUBTS				3.0	0.0	
TUBTS-Biotin				5.6	5.3	
OEG–TUBTS	12.8	28.4	56.0	2.8	0.0	
OEG–TUBTS–Biotin	5.5	24.8	61.9	4.6	3.1	
10–OEG–TFA	37.5	38.6	20.2			3.8
10–OEG	30.9	41.8	27.5			0.0
7–OEG–TFA	31.9	53.8	10.0			4.3
7–OEG	34.2	53.2	12.0			0.0
13–OEG–TFA	24.1	39.0	39.2			1.7
13–OEG	26.3	34.7	39.1			0.0
OEG–TUBTS/7–OEG–TFA	18.0	48.0	30.3	1.7	0.0	2.0
OEG–TUBTS/7–OEG–Biotin	22.7	36.5	35.8	2.5	2.5	0.0
						-
Si_3_N_4_/TUBTS	27.9	19.0	44.0	1.6	6.9	
Si_3_N_4_/TUBTS–Biotin	16.1	21.8	43.6	5.5	2.6	
						-
AlN/TUBTS	1.1	28.8	23.0	2.3	10.0	
AlN/TUBTS–Biotin	9.7	21.9	41.8	5.2	3.7	

^1^ Not all values were recorded.

## Data Availability

Data is contained within the article or supplementary materials.

## Supplementary Materials

Procedures for the synthesis of the molecules described in this paper together with attendant confirmatory analyses are available online at https://www.mdpi.com/1996-1944/14/2/472/s1.
