# Supplementary material for: Assembling Surface Linker Chemistry with Minimization of Non-Specific Adsorption on Biosensor Materials
Source: Materials (Basel). 2021 Jan 19;14(2):472. doi: 10.3390/ma14020472 (PMC7835736; doi:10.3390/ma14020472)
Supplement: Supplementary file 1 [file materials-14-00472-s001.zip › materials-1061115-supplementary.pdf]

materials

MDPI

Supplementary Materials

Assembling Surface Linker Chemistry with Minimization of Non-Specific Adsorption on Biosensor Materials

Jack Chih-Chieh Sheng, Brian De La Franier and Michael Thompson *

Department of Chemistry, University of Toronto, 80 St. George Street, Toronto, ON M5S 3H6, Canada; jack.sheng@mail.utoronto.ca (J.C.-C.S.); brian.delafranier@utoronto.ca (B.D.L.F.)
* Correspondence: m.thompson@utoronto.ca; Tel.: +1-416-978-3575

1. Synthesis of TUBTS

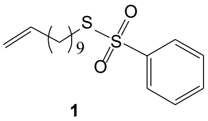

1

S-undec-10-enyl benzenethiosulfonate 1. To a stirred solution of 11-bromo-undec-1-ene (95%, 4.6 mL, 20.1 mmol, 1.0 equiv.) in MeCN (100 mL) were added benzenethionosulfonic acid sodium salt (85%, 9.2 g, 39.9 mmol, 2.0 equiv.) at rt. After overnight refluxing, the resulting solution was submitted to a EtOAc/H_2O extraction. The combined organic phases were then dried over anhydrous Na_2SO_4, filtered and finally evaporated *in vacuo*. Purification was achieved by column chromatography (Hexanes/EtOAc gradient 100/0 to 95/5) to afford 6.15 g (93%) of a pale cloudy yellow oil (> 95% purity). An additional careful column chromatography afforded pure **1** as a pale yellow oil. **1**: ^{1}H NMR (300 MHz, $CDCl_3$) δ 7.95 (m, 2H), 7.64 (m, 1H), 7.56 (m, 2H), 5.82 (m, 1H), 5.14-4.91 (m, 2H), 3.00 (t, J = 7.2 Hz, 2H), 2.04 (q, J = 7.0 Hz, 2H), 1.58 (qt, J = 7.2 Hz, 2H), 1.42-1.16 (m, 12H); ^{13}C NMR (75 MHz, $CDCl_3$) δ 145.2, 139.4, 133.7, 129.4, 127.2, 114.4, 36.3, 34.0, 29.5, 29.4, 29.2, 29.1, 29.0, 28.8, 28.7; IR (neat) 3070 cm^{-1}, 1640 cm^{-1}, 1328 cm^{-1}, 1147 cm^{-1}; HRMS (ESI, *m/z*) calcd. for $C_{17}H_{27}S_2O_2$ (MH^+) 327.1446, found 327.1447.

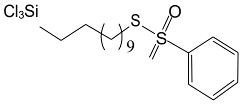

2

S-(11-trichlorosilyl-undecenyl) benzenethiosulfonate 2 (TUBTS). In a heavy-walled tube equipped with a magnetic stirring bar, **S-undec-10-enyl benzenethiosulfonate 1** (700 mg, 2.13 mmol, 1.0 equiv.) and $H_2PtCl_6.6H_2O$ (1 mg, 0.013 mmol, 0.1 mol. %) was placed. The tube was transferred into a glove-box and $HSiCl_3$ (0.30 mL, 2.94 mmol, 2.1 equiv.) was added to the solution. The tube was tightly fastened and then removed from the glove-box. The resulting solution was stirred at room temperature for 43 h behind a protecting shield. Excess $HSiCl_3$ was then removed under high vacuum to afford **2** as a viscous orange oil (633 mg, 95%). **2**: ^{1}H NMR (300 MHz, $CDCl_3$) δ 7.93 (m, 2H), 7.63 (m, 1H), 7.55 (m, 2H), 2.99 (t, J = 7.3 Hz, 2H), 1.65-1.51 (m, 4H), 1.44-1.16 (m, 16H).

Citation: Sheng, J.C.-C.; De La Franier, B.; Thompson, M. Assembling Surface Linker Chemistry with Minimization of Non-Specific Adsorption on Biosensor Materials. *Materials* **2021**, *14*, 472. https://doi.org/10.3390/ma14020472

Received: 17 December 2020
Accepted: 13 January 2021
Published: 19 January 2021

Publisher's Note: MDPI stays neutral with regard to jurisdictional claims in published maps and institutional affiliations.

Copyright: © 2021 by the authors. Licensee MDPI, Basel, Switzerland. This article is an open access article distributed under the terms and conditions of the Creative Commons Attribution (CC BY) license (http://creativecommons.org/licenses/by/4.0/).

2. Synthesis of OEG-TUBTS

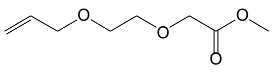

3

(2-allyloxy-ethoxy)-acetic acid methyl ester 3. To a stirred solution of 2-allyloxy-ethanol (5.5 mL, 50.0 mmol, 1.0 equiv.) in THF (50 mL) was carefully added NaH (60%, 2.4 g, 60.0 mmol, 1.2 equiv.) in small portions at rt. The solution was then refluxed for 1h (until H_2 release ceases). After cooling to 0ºC, the resulting solution is added dropwise to a stirred solution of Methyl bromoacetate (5.7 mL, 60.0 mmol, 1.2 equiv.) in THF (50 mL). After completion (30 min at 0ºC), the resulting solution was submitted to an EtOAc/H_2O extraction. The combined organic phases were washed with brine, dried over anhydrous Na_2SO_4, then filtered and evaporated *in vacuo*. Purification was achieved by column chromatography (Hexanes/EtOAc gradient 95/5 to 60/40). **3**: yellow oil (6.66 g, 70%); ^{1}H NMR (400 MHz, $CDCl_3$) δ 5.96-5.85 (m, 1H), 5.31-5.24 (m, 1H), 5.20-5.15 (m, 1H), 4.17 (s, 2H), 4.04-4.01 (m, 2H), 3.75 (s, 3H), 3.75-3.72 (m, 2H), 3.65-3.62 (m, 2H); ^{13}C NMR (100MHz, $CDCl_3$) δ 171.1, 134.8, 117.3, 72.4, 71.2, 69.7, 68.9, 51.9; IR (neat) 1755 cm^{-1}; HRMS (ESI, *m/z*) calcd. For $C_8H_{15}O_4$ (MH^+) 175.0964, found 175.0960.

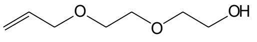

4

2-(2-allyloxy-ethoxy)-ethanol 4. To a stirred solution of ester **3** (9.77 g, 55.1 mmol, 1.0 equiv.) in THF (100 mL) was carefully added one portion of LAH (1.10 g, 27.5 mmol, 0.5 equiv.) at 0ºC. After 30 min, another portion of LAH was carefully added and then the reaction was stirred for an extra 30 min. The reaction was then carefully quenched with a dropwise addition of a Na_2SO_4 saturated aqueous solution. White aluminum salts were then filtered off over a short plug of Celite (EtOAc washings) and the resulting filtrate was finally evaporated *in vacuo*. Purification was achieved by distillation under reduced pressure using a Kugelrohr distillation apparatus to afforded **4** as a colourless oil (7.99 g, 99%). Spectroscopic data was consistent with those reported in the literature:[81] ^{1}H NMR (400 MHz, $CDCl_3$) δ 5.92 (ddt, J = 17.3, 10.4, 5.6 Hz, 1H), 5.29 (dq, J = 17.3, 1.6 Hz, 1H), 5.20 (dq, J = 10.4, 1.6 Hz, 1H), 4.04 (dt, J = 5.6, 1.6 Hz, 2H), 3.76-3.73 (m, 2H), 3.71-3.68 (m, 2H), 3.64-3.60 (m, 4H), 2.36 (brs, 1H).

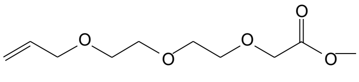

5

(2-(2-allyloxy-ethoxy)-ethoxy)-acetic acid methyl ester 5. To a stirred solution of alcohol **4** (4.86 g, 33.2 mmol, 1.0 equiv.) in THF (35 mL) was carefully added NaH (60%, 1.60 g, 40.0 mmol, 1.2 equiv.) in small portions at rt. The solution was then refluxed for 1h (until H_2 release ceases). After cooling to 0ºC, the resulting solution is added dropwise to a stirred solution of Methyl bromoacetate (5.7 mL, 60.0 mmol, 1.2 equiv.) in THF (35 mL). After completion (30 min at 0ºC), the resulting solution was submitted to an EtOAc/H_2O extraction. The combined organic phases were washed with brine, dried over anhydrous Na_2SO_4, and then filtered and evaporated *in vacuo*. Purification was achieved by distillation under high vacuum. **5**: colourless oil (5.49 g, 75%); bp = 130-140 0ºC (0.09 Torr); ^{1}H NMR (300 MHz, $CDCl_3$) δ 5.98-5.84 (m, 1H), 5.32-5.24 (m, 1H), 5.22-5.14 (m, 1H), 4.17 (s, 2H), 4.02 (m, 2H), 3.75 (s, 3H), 3.75-3.58 (m, 8H); ^{13}C NMR (75 MHz, $CDCl_3$) δ 171.0, 134.8, 117.1, 72.3, 71.0, 70.8, 70.7, 69.5, 68.7, 51.8; IR (neat) 1754 cm^{-1}; HRMS (ESI, *m/z*) calcd. for $C_{10}H_{19}O_5$ (MH^+) 219.1236, found 219.1227.

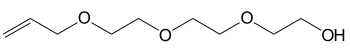

6

2-(2-(2-allyloxy-ethoxy)-ethoxy)-ethanol 6. To a stirred solution of ester **5** (4.60 g, 21.1 mmol, 1.0 equiv.) in THF (60 mL) was carefully added one portion of LAH (0.50 g, 12.5 mmol, 0.5 equiv.) at 0ºC. After 30 min, another portion of LAH was carefully added. The reaction was then stirred for an extra 30 min. The reaction was carefully quenched with a dropwise addition of a Na_2SO_4 saturated aqueous solution. White aluminum salts were then filtered off over a short plug of Celite (EtOAc washings) and the resulting filtrate was finally evaporated *in vacuo* to afford pure **6** (no purification required) as a pale yellow oil (3.89 g, 97%). Spectroscopic data were consistent with those reported in the literature:[81] ^{1}H NMR (300 MHz, $CDCl_3$) δ 5.93 (ddt, J = 17.2, 10.3, 5.7 Hz, 1H), 5.28 (dq, J = 17.2, 1.5 Hz, 1H), 5.19 (dq, J = 10.3, 1.5 Hz, 1H), 4.04 (dt, J = 5.7, 1.5 Hz, 2H), 3.78-3.58 (m, 12H), 2.51 (brs, 1H); ^{13}C NMR (75 MHz, $CDCl_3$) δ 134.8, 117.3, 72.7, 72.4, 70.8, 70.7, 70.5, 69.5, 61.8.

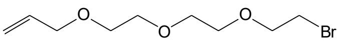

7

3-(2-(2-(2-bromo-ethoxy)-ethoxy)-ethoxy)-prop-1-ene 7. To a stirred solution of alcohol **6** (3.83 g, 20.1 mmol, 1.0 equiv.) and pyridine (0.16 mL, 2.00 mmol, 0.1 equiv.) in Et_2O (20 mL) was added dropwise phosphorus tribromide (0.74 mL, 7.60

mmol, 0.36 equiv.) at 0ºC. After 30 min at 0ºC, the reaction was allowed to warm to room temperature. As the reaction was apparently not complete after 12h of stirring, pyridine (1.60 mL, 20.0 mmol, 1.0 equiv.) and sodium bromide (4.14 g, 40.2 mmol, 2.0 equiv.) were added successively. After 12h of reflux, the resulting solution was submitted to an EtOAc/NH_4Cl saturated aqueous solution extraction. The combined organic phases were then dried over anhydrous Na_2SO_4, filtered and evaporated *in vacuo*. Purification was achieved by column chromatography (Hexanes/EtOAc gradient 95/5 to 60/40). **7**: yellow oil (0.90 g, 18%); ^{1}H NMR (300 MHz, $CDCl_3$) δ 5.93 (ddt, *J* = 17.3, 10.5, 5.7 Hz, 1H), 5.28 (dq, *J* = 17.3, 1.5 Hz, 1H), 5.19 (dq, *J* = 10.5, 1.5 Hz, 1H), 4.03 (dt, *J* = 5.7, 1.5 Hz, 2H), 3.82 (t, *J* = 6.3 Hz, 2H), 3.71-3.65 (m, 6H), 3.64-3.59 (m, 2H), 3.48 (t, *J* = 6.3 Hz, 2H); ^{13}C NMR (75 MHz, $CDCl_3$) δ 134.9, 117.4, 72.5, 71.4, 70.9, 70.8, 70.7, 69.6, 30.5.

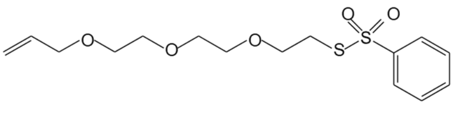

8

S-(2-(2-(2-allyloxy-ethoxy)-ethoxy)-ethyl) benzenethiosulfonate 8. To a stirred solution of bromide **7** (0.90 g, 3.6 mmol, 1.0 equiv.) in MeCN (18 mL) was added benzenethionosulfonic acid sodium salt (85%, 1.64 g, 7.1 mmol, 2.0 equiv.) at rt. After overnight refluxing, the resulting solution was submitted to an EtOAc/brine extraction. The combined organic phases were then dried over anhydrous Na_2SO_4, filtered and finally evaporated *in vacuo*. Purification was achieved by column chromatography (Hexanes/EtOAc gradient 95/5 to 30/70) to afford 1.04 g (84%) of a pale yellow oil (> 95% purity). An additional careful column chromatography afforded pure **8** as a pale yellow oil. **8**: ^{1}H NMR (300 MHz, $CDCl_3$) δ 7.95 (m, 2H), 7.65 (m, 1H), 7.56 (m, 2H), 5.91 (m, 1H), 5.32-5.14 (m, 2H), 4.01 (m, 2H), 3.72 (m, 10H), 3.20 (t, *J* = 6.3 Hz, 2H); ^{13}C NMR (75 MHz, $CDCl_3$) δ 145.0, 134.9, 133.9, 129.5, 127.2, 117.3, 72.4, 70.8, 70.7, 70.6, 69.6, 69.2, 35.9; IR (neat) 3068, 1647, 1324, 1142 cm^{-1}; HRMS (ESI, *m/z*) calcd. for $C_{15}H_{23}S_2O_5$ (MH^+) 347.0971, found 347.0981.

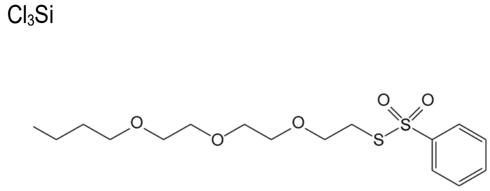

9

S-(2-(2-(2-(3-trichlorosilyl-propyloxy)-ethoxy)-ethoxy)-ethyl) benzenethiosulfonate 9 (OEG-TUBTS). In a heavy-walled tube equipped with a magnetic stirring bar, benzene-thiosulfonate **8** (347 mg, 1.00 mmol, 1.0 equiv.) and $H_2PtCl_6.6H_2O$ (1 mg, 0.002 mmol, 0.5 mol. %) were placed. The tube was transferred into a glove-box and $HSiCl_3$ (0.30 mL, 2.94 mmol, 3.0 equiv.) was added to the solution. The tube was tightly fastened and then removed from the glove-box. The resulting solution was stirred at room temperature for 21 h behind a protecting shield. $HSiCl_3$ excess was then removed under high vacuum to afford **9** as a viscous yellow- orange cloudy oil (444 mg, 92%). **9**: ^{1}H NMR (300 MHz, $CDCl_3$) δ 7.94 (m, 1H), 7.62 (m, 1H), 7.56 (m, 1H), 7.46 (m, 1H), 7.32 (m, 1H), 3.85-3.52 (m, 16H), 3.20 (t, *J* = 6.2 Hz, 1H), 2.80 (t, *J* = 6.2 Hz, 1H).

3. Synthesis of 10-OEG-TFA

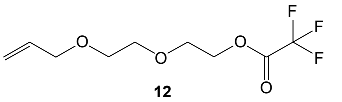

12

Trifluoroacetic acid 2-(2-allyloxy-ethoxy)-ethyl ester 12. To a stirred solution of alcohol **4** (2.92 mL, 20.0 mmol, 1.0 equiv.), triethylamine (5.6 mL, 40.0 mmol, 2.0 equiv.) and 4-DMAP (0.25 g, 2.0 mmol, 0.1 equiv.) in CH_2Cl_2 (40 mL) was added dropwise trifluoroacetic anhydride (3.37 mL, 24.0 mmol, 1.2 equiv.) at 0ºC. The solution was then allowed to warm to room temperature and then stirred overnight. The resulting solution was submitted to a CH_2Cl_2/saturated NH_4Cl aqueous solution extraction. The combined organic phases were dried over anhydrous Na_2SO_4, filtered then evaporated *in vacuo*. Purification was achieved by distillation under reduced pressure. **12**: colourless oil (3.61 g, 70%); bp = 100-110ºC (water tap vacuum); ^{1}H NMR (400 MHz, $CDCl_3$) δ 5.91 (ddt, *J* = 17.2, 11.6, 5.6 Hz, 1H), 5.27 (dq, *J* = 17.2 Hz, 1H), 5.18 (dq, *J* = 11.6, 1H), 4.50 (t, *J* = 4.8 Hz, 2H), 4.02 (dt, *J* = 5.6 Hz, 2H), 3.80 (t, *J* = 4.8 Hz, 2H), 3.64 (m, 4H); ^{13}C NMR (100 MHz, $CDCl_3$) δ 157.8 (q, *J* = 42.4 Hz), 134.2, 117.7, 114.8 (q, *J* = 283.9Hz) 72.2, 71.0, 68.7, 67.8 66.2.

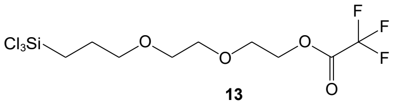

13

Trifluoroacetic acid 2-(2-(3-trichlorosilyl-propyloxy)-ethoxy)-ethyl ester 15 (10-OEG-TFA). In a heavy-walled tube equipped with a magnetic stirring bar, OEG-diluent precursor **12** (0.50 g, 1.9 mmol, 1.0 equiv.) and H2PtCl6.6H2O (1 mg, 0.002 mmol, 0.1 mol. %) were placed. The tube was transferred into a glove-box and HSiCl3 (0.39 mL, 3.8 mmol, 2.0 equiv.) was added to the solution. The tube was tightly fastened then removed from the glove-box. The resulting solution was stirred at room temperature for 20 h behind a protecting shield. HSiCl3 excess was then removed under high vacuum to afford **13** as an orange-brown oil (0.67 g, 90%). **13**: ^{1}H NMR (400 MHz, CDCl3) δ 4.50 (m, 2H), 3.79 (m, 2H), 3.65 (m, 4H), 3.51(m, 2H), 1.85 (m, 2H), 1.47 (m, 2H); ^{13}C NMR (100 MHz, CDCl3) δ 157.6 (q, *J* = 42.4 Hz), 114.2 (q, *J* = 284.0 Hz), 71.8, 70.6, 70.2, 68.1, 66.5, 22.2, 20.8.

4. Synthesis of 7-OEG-TFA

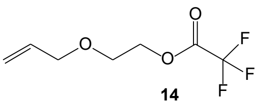

14

Trifluoroacetic acid 2-allyloxy-ethyl ester 10. To a stirred solution of 2-allyloxy-ethanol (4.36 mL, 40.0 mmol, 1.0 equiv.), triethylamine (11.2 mL, 80.0 mmol, 2.0 equiv.) and 4-DMAP (0.49 g, 4.0 mmol, 0.1 equiv.) in CH_2Cl_2 (80 mL) was added dropwise trifluoroacetic anhydride (6.74 mL, 48.0 mmol, 1.2 equiv.) at 0ºC. The solution was then allowed to warm to room temperature then stirred overnight. The resulting solution was submitted to a CH_2Cl_2/saturated NH_4Cl aqueous solution extraction. The combined organic phases were dried over anhydrous Na_2SO_4, filtered then evaporated *in vacuo*. Purification was achieved by distillation under reduced pressure. **10**: colourless oil (5.76 g, 72%); bp = 72-74ºC (water tap vacuum); ^{1}H NMR (400 MHz, CDCl3) δ 5.88 (m, 1H), 5.29 (brd, *J* = 17.2 Hz, 1H), 5.20 (m, 1H), 4.50 (t, *J* = 4.8 Hz, 2H), 4.03 (m, 2H), 3.73 (t, *J* = 4.8 Hz, 2H); ^{13}C NMR (75 MHz, CDCl3) δ 157.7 (q, *J* = 4.1 Hz), 134.2, 117.8, 114.7 (q, *J* = 283.9 Hz), 72.4, 67.2, 67.0.

15. Trifluoroacetic acid 2-(3-trichlorosilyl-propyloxy)- ethyl ester (7-OEG-TFA).

In a heavy-walled tube equipped with a magnetic stirring bar, OEG-diluent precursor **10** (3.97 g, 20.0 mmol, 1.0 equiv.) and $H_2PtCl_6.6H_2O$ (104 mg, 0.20 mmol, 1.0 mol. %) were placed. The tube was transferred into a glove-box and $HSiCl_3$ (4.10 mL, 40.2 mmol, 2.0 equiv.) was added to the solution. The tube was tightly fastened then removed from the glove-box. The resulting solution was stirred at room temperature for 20 h behind a protecting shield. Purification was achieved by Kugelrohr distillation under high vacuum. **11**: colourless oil (5.46 g, 82%); bp = 115-120 ºC (0.09 Torr); ^{1}H NMR (400 MHz, $CDCl_3$) δ 4.50 (m, 2H), 3.73 (m, 2H), 3.54 (t, *J* = 6.2 Hz, 2H), 1.85 (m, 2H), 1.48 (m, 2H); ^{13}C NMR (100 MHz, $CDCl_3$) δ 157.7 (q, *J* = 42.4 Hz), 114.3 (q, *J* = 284.0 Hz), 71.8, 67.9, 67.0, 22.8, 21.0.

5. Synthesis of 13-OEG-TFA

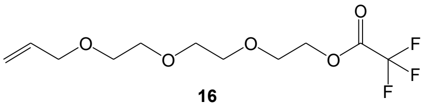

16

Trifluoroacetic acid 2-(2-(2-allyloxy-ethoxy)-ethoxy)-ethyl ester 16. To a stirred solution of alcohol **6** (1.92 mL, 10.0 mmol, 1.0 equiv.), triethylamine (2.8 mL, 20.0 mmol, 2.0 equiv.) and 4-DMAP (0.13 g, 1.0 mmol, 0.1 equiv.) in CH_2Cl_2 (20 mL) was added dropwise trifluoroacetic anhydride (1.69 mL, 12.0 mmol, 1.2 equiv.) at 0ºC. The solution was then allowed to warm to room temperature then stirred overnight. The resulting solution was submitted to a CH_2Cl_2/saturated NH_4Cl aqueous solution extraction. The combined organic phases were dried over anhydrous Na_2SO_4, filtered then evaporated *in vacuo*. Purification was achieved by distillation under high vacuum then followed by a quick column chromatography (CH_2Cl_2/EtOAc gradient 100/0 to 95/5).

16: pale yellow oil (1.72 g, 60%); bp = 140-150ºC (water tap vacuum); ^{1}H NMR (400 MHz, $CDCl_3$) δ 5.91 (*J* = 17.2, 10.4, 5.6 Hz, 1H), 5.27 (dq, *J* = 17.2, 1.6 Hz, 1H), 5.17 (dq, *J* = 10.4, 1.6 Hz, 1H) 4.49 (t, *J* = 4.8 Hz, 2H), 4.02 (dt, *J* = 5.6 Hz, 2H), 3.79 (t, *J* = 4.8 Hz, 2H), 3.69-3.58 (m, 8H); ^{13}C NMR (100 MHz, $CDCl_3$) δ 157.8 (q, *J* = 42.4 Hz), 134.3, 117.5, 114.8 (q, *J* = 283.8Hz) 72.2, 70.8, 70.7, 70.6, 69.4, 68.2, 67.0.

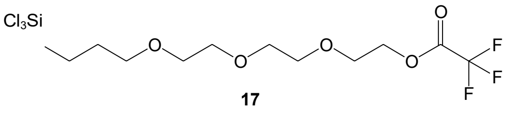

17

Trifluoroacetic acid 2-(2-(2-(3-trichlorosilyl-propyloxy)-ethoxy)-ethoxy)-ethyl ester 17 (13-OEG-TFA).

In a heavy-walled tube equipped with a magnetic stirring bar, OEG-diluent precursor **16** (0.50 g, 1.7 mmol, 1.0 equiv.) and $H_2PtCl_6.6H_2O$ (1 mg, 0.002 mmol, 0.1 mol. %) were placed. The tube was transferred into a glove-box and $HSiCl_3$ (0.35 mL, 3.4 mmol, 2.0 equiv.) was added to the solution. The tube was tightly fastened then removed from the glove-box. The resulting solution was stirred at room temperature for 20 h behind a protecting shield. $HSiCl_3$ excess was then removed under high vacuum to afford **17** as an orange-brown oil (0.64 g, 90%). **17**: ^{1}H NMR (400 MHz, $CDCl_3$) δ 4.47 (m, 2H), 3.77 (m, 2H). 3.70-3.47 (m, 10H), 1.85 (m, 2H), 1.47 (m, 2H); ^{13}C NMR (100 MHz, $CDCl_3$) δ 157.8 (q, *J* = 42.6 Hz), 114.4 (q, *J* = 283.7Hz) 71.9, 70.8, 70.7, 70.6, 68.2, 67.0, 65.9, 22.4, 21.0.
